# Administration of breast milk cell fractions to neonates with birthweight equal to or less than 1800 g: a randomized controlled trial

**DOI:** 10.1186/s13006-021-00405-0

**Published:** 2021-08-23

**Authors:** Minoo Fallahi, Seyed Masoud Shafiei, Naeeme Taslimi Taleghani, Maryam Khoshnood Shariati, Shamsollah Noripour, Fatemeh Pajouhandeh, Sina Kazemian, Mahmood Hajipour, Mohammad Kazemian

**Affiliations:** 1 Neonatal Health Research Center, Research Institute for Children’s Health, Shahid Beheshti University of Medical Sciences, Tehran, Iran; 2 Department of Neonatology, Mahdieh Maternity Hospital, Shahid Beheshti University of Medical Sciences, Tehran, Iran; 3 Students’ Scientific Research Center (SSRC), Tehran University of Medical Sciences, Tehran, Iran; 4 Department of Epidemiology, School of Public Health and Safety, Shahid Beheshti University of Medical Sciences, Tehran, Iran

**Keywords:** Breastfeeding, Breast milk cell, Neonate, Neonatal mortality, Preterm infants

## Abstract

**Background:**

Most premature and very low birthweight infants cannot tolerate breast milk feeding in the first few days of life and are deprived of its benefits. This study evaluates the clinical outcomes of administering breast milk cell fractions to neonates with a birthweight of ≤1800 g.

**Methods:**

We conducted a randomized controlled trial on 156 infants in the neonatal intensive care unit of Mahdieh Maternity Hospital in Tehran, Iran, from May 2019 to April 2020. All neonates with a birthweight ≤1800 g were enrolled and divided into intervention and control groups using stratified block randomization. Neonates in the intervention group received the extracted breast milk cell fractions (BMCFs) of their own mother’s milk after being centrifuged in the first 6 to 12 h after birth. The control group received routine care, and breastfeeding was started as soon as tolerated in both groups. Study outcomes were necrotizing enterocolitis (NEC), death, and in-hospital complications.

**Results:**

We divided participants into two groups: 75 neonates in the intervention group and 81 neonates in the control group. The mean birthweight of neonates was 1390.1 ± 314.4 g, and 19 (12.2%) neonates deceased during their in-hospital stay. The incidence of NEC was similar in both groups. After adjustment for possible confounders in the multivariable model, receiving BMCFs were independently associated with lower in-hospital mortality (5 [26.3%] vs. 70 (51.1%]; odds ratio (OR): 0.24; 95% confidence interval [CI] 0.07, 0.86). Also, in a subgroup analysis of neonates with birthweight less than 1500 g, in-hospital mortality was significantly lower in the intervention group (4 [9.5%] vs. 13 [30.2%]; OR: 0.24; 95% CI 0.07, 0.82). There were no differences in major complications such as bronchopulmonary dysplasia and retinopathy of prematurity between the two groups. No adverse effects occurred.

**Conclusions:**

Our research demonstrated a significantly lower mortality rate in neonates (with a birthweight of ≤1800 g) who received breast milk cell fractions on the first day of life. Since this is a novel method with minimal intervention, we are looking forward to developing and evaluating this method in larger studies.

**Trial registration:**

IIranian Registry of Clinical Trials. Registered 25 May 2019, IRCT20190228042868N1.

## Background

Breast milk is the best source of nutrition for all babies, including premature infants. It plays a notable and sensitive role in improving the function of the immune system [1]. It contains lipids, proteins, carbohydrates, and bioactive molecules, such as vitamins, immunomodulatory factors, and several different types of mediators [2, 3]. The immunological properties of breast milk differ during breastfeeding periods. Thus, breast milk’s nutritional content is compatible with neonatal requirements. According to recent studies, breast milk components can change in response to neonatal infection, and some proved that breast milk fractions might enhance the transepithelial absorption of extrinsic iron (non-milk iron) and its bioavailability [4–6]. Breast milk prevents diseases related to free oxygen radicals due to its potent antioxidant property; some examples are necrotizing enterocolitis (NEC), retinopathy of prematurity (ROP), and bronchopulmonary dysplasia (BPD) [7–9].

We cannot initiate feeding in some preterm (gestational age < 37 weeks) and very low birthweight (birthweight < 1500 g) babies in the first few days of life due to limitations like respiratory distress, poor sucking, swallowing, low gastrointestinal motility, and lack of digestive enzymes [10–12]. Despite the strong recommendation on early feeding and minimal enteral nutrition in the first days of life, it is postponed in some sick preterm neonates for several days or weeks after birth, and these infants are deprived of this optimum source of nutrition. We can reduce the outcomes of delayed-onset breastfeeding by starting early enteral feeding with a minimum tolerable amount of breast milk in preterm infants and reach full breast milk feeding earlier [10, 11].

A study by Maffei et al. [13] investigated early oral colostrum administration in preterm neonates and infants who received the oral colostrum by syringe had significantly higher urinary secreted immunoglobulin A and lactoferrin comparing to application with a swab. Many studies have suggested early breastfeeding initiation (< 24 h) leads to a decrease in neonatal mortality [13, 14]. However, there are few controlled trials about the early progressive feeding regarding preterm infants.

Breast milk has different fractions after centrifugation at 4 °C; it consists of an upper-fat fraction, and the rest is skimmed milk, a combination of casein, whey, and cellular strains with numerous types of cells that precipitate [6]. Recent studies have discovered mesenchymal cells, including progenitor cells, stem cells, and myoepithelial cells, as well as different bioactive factors during the first 6 months of breastfeeding in widespread concentration ranges. Yet, we still do not know or understand the relationship between these bioactive factors, mesenchymal stem cell content, and their health implications. Some studies suggest an essential role in organogenesis, anti-viral protection, and not only short-term effects but also long-term benefits for these components in breast milk cell fractions [4, 15–19]. In this trial, we separated the creamy layer from the expressed breast milk, and the neonates received cellular and immune fractions of breast milk during the first 6–12 h after birth, and compared them to other babies who received routine care in the ward.

## Methods

### Study design and participants

This randomized controlled trial was conducted at Mahdieh maternity Hospital affiliated with Shahid Beheshti University of Medical Sciences in Tehran, Iran, from May 2019 to April 2020. The inclusion criteria were all inborn neonates with a birthweight of ≤1800 g. The exclusion criteria were significant congenital anomalies, severe birth asphyxia (APGAR score < 3 at the first minute), other lethal diseases during the follow-up period (such as severe metabolic diseases, massive intracranial hemorrhage, etc.), and mothers with conditions that contraindicated breast milk feeding. The primary outcome was infants diagnosed with NEC during the hospital course, and we also followed up all infants who incurred death and in-hospital complications.

### Breast milk cell fractions sampling and intervention

**Fig. 1:**
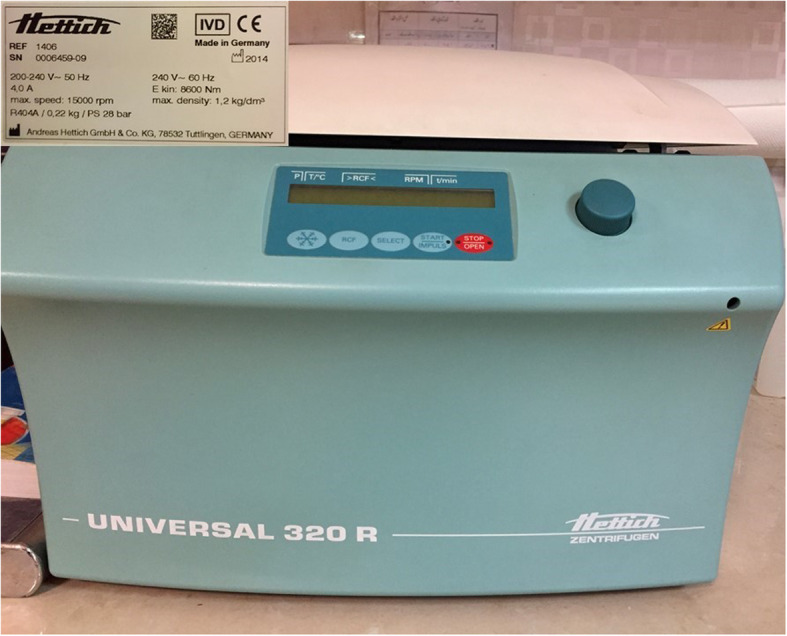
Refrigerated centrifuge“ Universal 320R Hettich Zentrifugen”

**Fig. 2:**
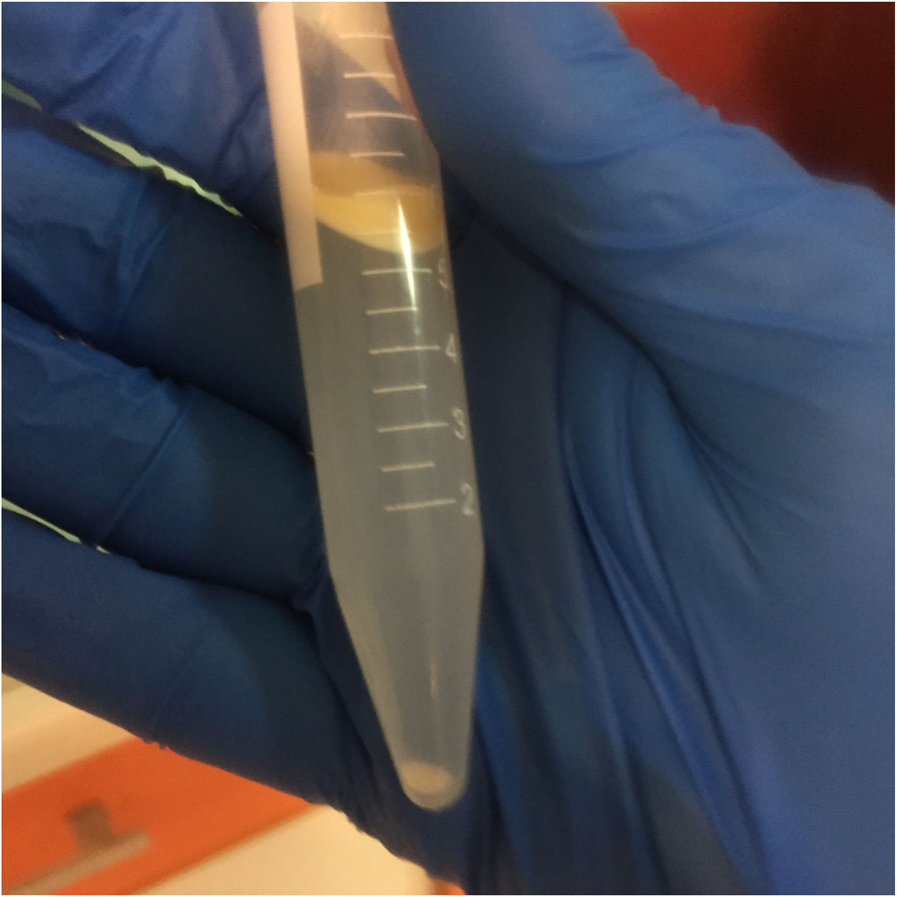
Centrifuged breast milk sample. A creamy layer on the top, followed by a watery phase with cream-colored strings at the bottom of the sample that contains most of the cells

All mothers were asked to express their breast milk within the first 6 hours after birth. A refrigerated centrifuge was used to separate the fresh expressed break milk components at a speed of 600 rounds per minute for 5 minutes at 4 °C temperature (Fig. 1). During the preparation procedure, after centrifuging 10–12 ml of breast milk in the refrigerated centrifuge “Universal 320 R Hettich Zentrifugen” a creamy layer starts to form on top of the sample tube while the cold helps it to stick together (Fig. 2). Then a large part of this material was removed using one or two sterile cotton swaps so the Pasteur pipette or insulin syringe (without needle) could easily reach the inside the tube. At that time, 0.1–0.2 ml of the lower semi-solid (watery) part and cream-colored strings at the bottom of the tube were dropped in the oral cavity of the neonates who participated in the intervention group during the first 6–12 h after birth. Hygiene protocols were considered in all steps of milk preparation. Both groups received all neonatal intensive care unit routine care, and breast milk initiation was based on the neonates’ general condition. Blinding was not possible due to the nature of the intervention. Nevertheless, the participants were unaware of the randomization list but not blinded to the study group. The potential risk of aspiration was considered as an adverse event for this intervention, while we did not record any adverse effects during this trial.

### Data collection and definitions

Data were collected from hospital medical records. Demographics and clinical outcomes were recorded using a pre-prepared checklist. All participants were observed for infants diagnosed with NEC, BPD, ROP, early-onset sepsis (positive blood culture), hospital length of stay, and in-hospital mortality. NEC, BPD, and ROP were defined using Bell’s criteria, the National Institute of Health consensus definition, and international classification of retinopathy of prematurity consecutively [20–22].

### Sample size and statistical analysis

The sample size was calculated to detect a 20% difference in NEC morbidity rate after using breast milk cell fractions, assuming an 11% NEC morbidity rate [23]. We used the sample size calculation for comparing proportions [24], considering a 5% alpha-type error rate and a statistical power of 80%. After correcting the 15% sample volume loss in each group, a sample size of 80 participants was needed for each group. Neonates were 1:1 randomly assigned to the case and control group using a computer-generated block randomization sequence of variable block-sized [25], stratified for birthweight of 1800 g. According to the stratified block randomization method, patients were divided into two equal groups: a) neonates with birthweight less than 1500 g and b) neonates with birthweight 1500–1800 g.

$$ N={\left({\mathrm{Z}}_{\upalpha /2}+{\mathrm{Z}}_{\upbeta}\right)}^2\ast \left({\mathrm{P}}_1\left(1-{\mathrm{P}}_1\right)+{\mathrm{P}}_2\left(1-{\mathrm{P}}_2\right)\right)/{\left({\mathrm{P}}_1-{\mathrm{P}}_2\right)}^2 $$

Where for the sample size N, Z_α/2_ was the critical value of the normal distribution at α/2 (considering a 5% alpha-type error rate, the critical value is 1.96), Z_β_ was the critical value of the normal distribution at β (considering a statistical power of 80%, the critical value is 0.84) and *p*_1_ and *p*_2_ were the expected sample proportions of the two groups [24].

Categorical variables were presented as numbers (percentage) and compared using chi-square and Fisher exact test. Numerical variables are reported as mean ± standard deviation, and the Kolmogorov-Smirnov test was used to evaluate the distribution. Means of numerical variables were compared using an independent group T-test if the data were normally distributed; otherwise, the Mann–Whitney U-test was used. We fitted binary logistic regression analysis to evaluate risk factors associated with in-hospital mortality. In this study, all numerical variables had non-normal distribution. Variables with *P*-value < 0.1 in the univariate model (including gestational age, birthweight, and 1-min APGAR score) were considered as possible confounders and entered the multivariable model. We employed a standard entry method to adjust these models for possible confounders. The Hosmer–Lemeshow’s test was used to evaluate the goodness of fit for logistic regression models. Data were analyzed using SPSS version 23, and *P*-value < 0.05 is considered statistically significant.

## Results

**Fig. 3:**
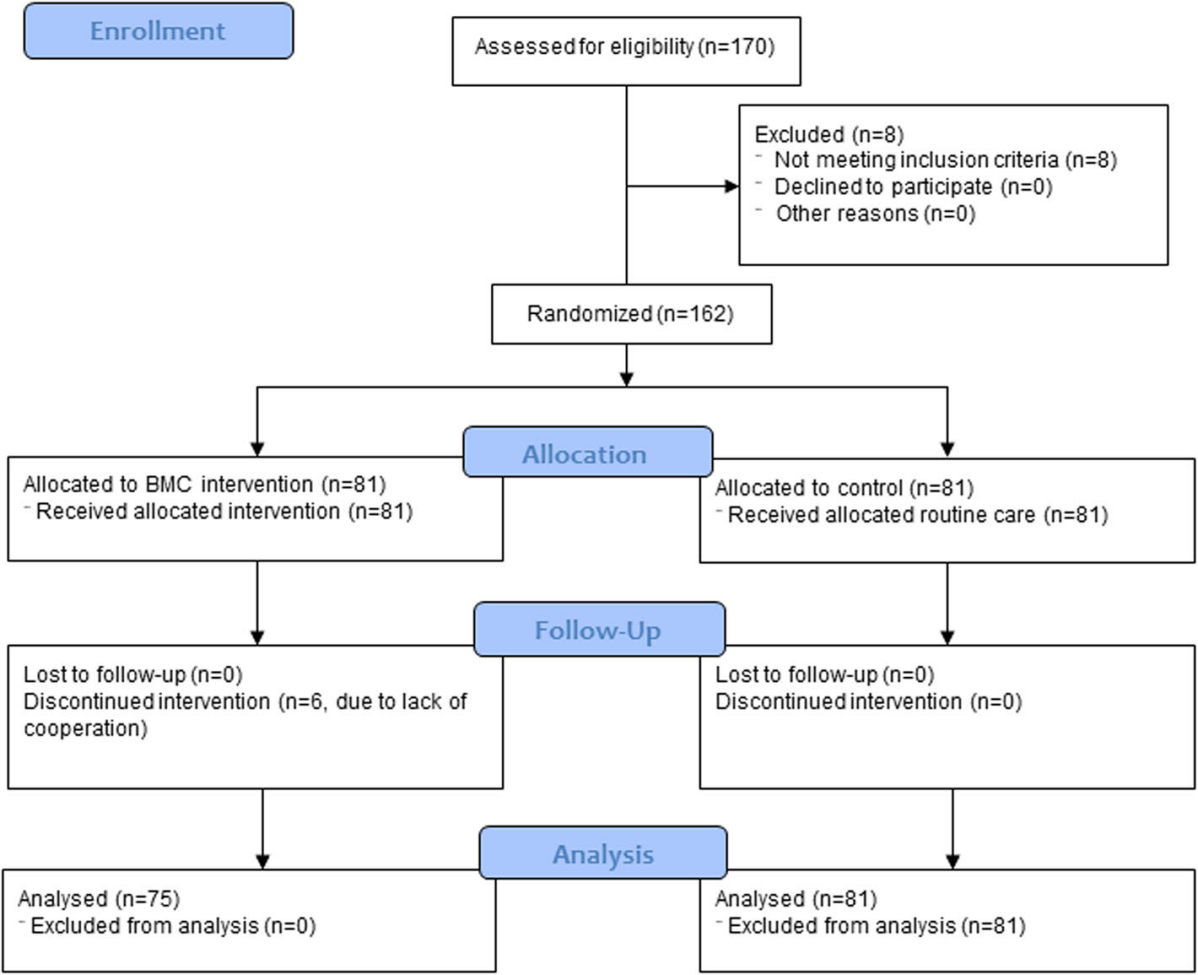
CONSORT study flow chart

Based on the study protocol, 170 neonates were eligible for enrollment into the study. We excluded eight neonates who did not meet the inclusion criteria, and six infants in the intervention group were excluded during the follow-up due to lack of cooperation. Eventually, 156 neonates with a birthweight of ≤1800 g entered the final analysis, including 75 neonates in the intervention group who received breast milk cell fractions (BMCFs) and 81 neonates who received routine care as the control group (Fig. 3). The birthweight mean was 1390.1 ± 314.4 g. Also, 69 (44%) infants were female, and mode of delivery births was normal vaginal delivery in 33 (21.2%). The incidence rate of NEC was 7.1% (11 out of 156), with an overall mortality rate of 12.2%.

The baseline characteristics, in-hospital outcomes, and neonatal complications between intervention and control groups are presented in (Table.1). Demographics and baseline characteristics were similar between two groups except for higher C/S birth in the intervention group (65 [86.7%] vs. 58 [71.6%]; OR: 2.58; 95% CI 1.13, 5.87; *P*-value: 0.02). During this study, one infant fed only with infant formula milk, and 19 infants used formula milk as a supplement and then switched to breast milk feeding. The infant formula milk consumption during hospitalization was lower in the intervention group (6 [8.0%] vs. 14 [17.3%]; OR: 0.42; 95% CI 0.15, 1.15; *P*-value: 0.08), but the difference was not significant. No infant with formula milk consumption was deceased during the in-hospital course. There was a weak evidence for lower incidence of NEC (4 [5.3%] vs. 7 [8.8%]; OR: 0.59; 95% confidence interval [CI] 0.16, 2.09; *P*-value: 0.41) in the intervention group, but the difference was not significant. In-hospital mortality was significantly lower in the intervention group compared to the control group (5 [6.7%] vs. 14 [17.3%]; Odds ratio [OR]: 0.34; 95% CI 0.12, 1.00; *P*-value: 0.04). We observed weak evidence for less positive blood cultures (4 [5.4%] vs. 6 [7.6%]; OR: 0.69; 95% CI 0.19, 2.57; *P*-value: 0.58), and shorter intubation period (3.2 ± 7.1 days vs. 4.0 ± 6.6 days; Degree of freedom (Df): 152; *P*-value: 0.17) in neonates in the intervention group, but the difference was not significant. The in-hospital complications were similar in both groups. Infants in the control group who deceased were younger than those in the BMCFs group (12.9 ± 8.9 days vs. 21.4 ± 32.8 days; Df: 4.21; *P*-value: 0.60), but the difference was not significant.

**Table 1:** Baseline characteristics and clinical outcomes in neonates who received BMCFs and the control group

Characteristic*	Total (***N*** = 156)	Received BMCFs (***N*** = 75)	Control (***N*** = 81)	OR (95% CI)‡	Df ‡	P †
Gestational age (week)	30.79 ± 2.47	31.00 ± 2.61	30.59 ± 2.34		151	0.18
Birthweight (grams)	1390.1 ± 314.4	1392.0 ± 325.6	1388.3 ± 305.7		154	0.85
Sex						
Female	69 (44.2%)	34 (45.3%)	35 (43.2%)	1.09 (0.58, 2.05)		0.79
Male	87 (55.8%)	41 (54.7%)	46 (56.8%)			
Delivery mode						
NVD	33 (21.2%)	10 (13.3%)	23 (28.4%)	2.58 (1.13, 5.87)		**0.02**
C-Section	123 (78.8%)	65 (86.7%)	58 (71.6%)			
1-min Apgar	8.0 [6.0–8.0]	9.0 [8.0–10.0]	9.0 [8.0–9.0]		154	0.53
5-min Apgar	9.0 [8.0–9.75]	9.0 [8.0–10.0]	9.0 [8.0–9.0]		154	0.42
Gravidity	2.0 [1.0–3.0]	2.0 [1.0–3.0]	2.0 [1.0–3.0]		154	0.91
Parity	1.0 [1.0–2.0]	1.0 [1.0–2.0]	1.0 [1.0–2.0]		154	0.52
**Use of infant formula**						
Formula milk consumption	20 (12.8%)	6 (8.0%)	14 (17.3%)	0.42 (0.15, 1.15)		0.08
Formula milk consumption duration (day)	17.4 ± 12.7	14.8 ± 11.4	18.5 ± 13.4		18	0.57
**In-hospital outcomes**						
Hospital length of stay (day)	28.0 ± 22.9	29.8 ± 23.8	26.3 ± 21.9		154	0.48
Discharge weight (grams)	1694.6 ± 212.6	1679.1 ± 195.2	1710.8 ± 229.7		135	0.60
Mortality	19 (12.2%)	5 (6.7%)	14 (17.3%)	0.34 (0.12, 1.00)		**0.04**
Intubation period (day)	3.6 ± 6.9	3.2 ± 7.1	4.0 ± 6.6		152	0.17
NIV period (day)	8.1 ± 10.9	9.4 ± 11.9	6.9 ± 9.7		153	0.21
NEC	11 (7.1%)	4 (5.3%)	7 (8.8%)	0.59 (0.16, 2.09)		0.41
BPD	24 (15.4%)	14 (18.7%)	10 (12.3%)	1.63 (0.67, 3.93)		0.27
ROP	41 (26.3%)	21 (28.4%)	20 (24.7%)	1.21 (0.59, 2.47)		0.60
Positive blood culture	10 (6.4%)	4 (5.4%)	6 (7.6%)	0.69 (0.19, 2.57)		0.58

* Data are presented as mean ± standard deviation, number (%), or median [interquartile range]

† Statistically significant *P*-values are bolded. Categorical variables were compared using the chi-square test and numerical variables were compared using the Mann–Whitney U-test

‡ Odds ratio (OR) and 95% confidence interval (95% CI) are presented for categorical variables, and the degree of freedom (df) is presented for numerical variables

*BMCFs* breast milk cell fractions, *BPD* bronchopulmonary dysplasia, *C-Section* Cesarean section, *DF* degree of freedom, *NEC* necrotizing enterocolitis, *NIV* non-invasive ventilation, *NVD* normal vaginal delivery, *OR* odds ratio, *ROP* retinopathy of prematurity

In a subgroup analysis, we separately compared the in-hospital outcomes of both intervention and control groups in two categories: a) neonates with a birthweight of less than 1500 g and b) neonates with a birthweight of 1500–1800 g (Table 2). The incidence of NEC was similar between the intervention and control in both subgroups. About neonates with a birthweight < 1500 g, there was a significantly lower mortality rate in the intervention group compared to the control group (4 [9.5%] vs. 13 [30.2%]; OR: 0.24; 95% CI 0.07, 0.82; *P*-value: 0.02). However, in neonates with a 1500–1800 g birthweight, the mortality rate was similar between the two groups (*P*-value: 0.92). In terms of in-hospital complications, there was no statistically significant difference between the two groups. Nevertheless, there was weak evidence for the lower incidence of less positive blood cultures and shorter intubation periods in the intervention group in both subgroups, which is in line with our prior results.

**Table 2:** In-hospital outcomes according to the birthweight in neonates who received BMCFs and the control group

In-hospital outcomes*	Birthweight 1500-1800 g		OR (95% CI)‡	DF‡	P †	Birthweight < 1500 g		OR (95% CI)‡	DF ‡	P †
	Received BMCFs (***N*** = 33)	Control (***N*** = 38)				Received BMCFs (***N*** = 42)	Control (***N*** = 43)			
Hospital length of stay (day)	14.7 ± 7.1	18.3 ± 13.6		69	0.35	41.6 ± 25.7	33.4 ± 25.4		83	0.09
Discharge weight (grams)	1683.7 ± 113.6	1683.6 ± 166.5		67	0.38	1675.1 ± 245.4	1744.3 ± 289.1		66	0.15
Mortality	1 (3.0%)	1 (2.6%)	1.16 (0.07, 19.24)		0.92	4 (9.5%)	13 (30.2%)	0.24 (0.07, 0.82)		**0.02**
Intubation period (day)	1.2 ± 1.5	1.5 ± 2.8		67	0.82	4.7 ± 9.0	6.2 ± 8.2		83	0.07
NIV period (day)	3.5 ± 2.7	4.2 ± 4.0		69	0.64	14.1 ± 14.3	9.3 ± 12.4		82	0.06
NEC	1 (3.0%)	2 (5.3%)	0.56 (0.05, 6.50)		0.64	3 (7.1%)	5 (11.9%)	0.57 (0.13, 2.55)		0.46
BPD	0	2 (5.3%)	–		0.18	14 (33.3%)	8 (18.6%)	2.19 (0.80, 5.95)		0.12
ROP	1 (3.0%)	3 (7.9%)	0.36 (0.04, 3.68)		0.37	20 (48.8%)	17(39.5%)	1.46 (0.61, 3.46)		0.39
Positive blood culture	1 (3.0%)	1 (2.7%)	1.12 (0.07, 18.73)		0.93	3 (7.3%)	5 (11.9%)	0.58 (0.13, 2.62)		0.48

* Data are presented as mean ± standard deviation, number (%)

† Statistically significant *P*-values are bolded. Categorical variables were compared using the chi-square test and numerical variables were compared using the Mann–Whitney U-test

‡ Odds ratio (OR) and 95% confidence interval (95% CI) are presented for categorical variables, and the degree of freedom (DF) is presented for numerical variables

*BMCFs* breast milk cell fractions, *BPD* bronchopulmonary dysplasia, *DF* degree of freedom, *NEC* necrotizing enterocolitis, *NIV* non-invasive ventilation, *OR* odds ratio, *ROP* retinopathy of prematurity

We used binary logistic regression analysis to evaluate the predisposing factors associated with NEC and in-hospital mortality (Table.3). In univariate regression model, administration of BMCFs did not decrease the incidence of NEC (OR: 0.59; 95% CI 0.16, 2.09; *P*-value: 0.41). On the other hand, higher gestational age, higher birthweight, higher 1-min APGAR score, and receiving BMCFs were significantly associated with lower in-hospital mortality based on the univariate model. In multivariate model, after adjustment with these variables, only higher birthweight (OR:0.99; 95% CI 0.99, 1.00; *P*-value < 0.01) and receiving BMCFs (OR: 0.24; 95% CI 0.07, 0.86; *P*-value: 0.03) were independently associated with lower in-hospital mortality. Hosmer and Lemeshow’s test demonstrate that the model fits the data well (*P*-value: 0.56).

**Table 3:** In-hospital mortality logistic regression models

Characteristic*	Deceased (***N*** = 19)	Survived (***N*** = 137)	Univariate model		Multivariate model ‡	
			OR (95% CI)	***P*** †	OR (95% CI)	***P*** †
**Demographics**						
Gestational age (week)	28.70 ± 2.62	31.08 ± 2.31	0.95 (0.92, 0.97)	**< 0.01**	0.99 (0.95, 1.03)	0.59
Birthweight (grams)	1045.8 ± 269.9	1437.8 ± 290.1	0.99 (0.99, 1.00)	**< 0.01**	0.99 (0.99, 1.00)	**< 0.01**
Sex						
Female	11 (57.9%)	58 (42.3%)	1.87 (0.71, 4.95)	0.21		
Male	8 (42.1%)	79 (57.7%)				
**Delivery parameters**						
Delivery mode						
NVD	5 (26.3%)	28 (20.4%)	0.72 (0.24, 2.17)	0.56		
C-Section	14 (73.7%)	109 (79.6%)				
1-min Apgar	7.0 [5.0–8.0]	8.0 [6.0–8.0]	0.73 (0.54, 0.98)	**0.04**	1.08 (0.71, 1.64)	0.72
5-min Apgar	9.0 [8.0–9.0]	9.0 [8.0–10.0]	0.68 (0.46, 1.02)	0.06		
Gravidity	2.0 [1.0–3.0]	2.0 [1.0–3.0]	1.06 (0.74, 1.52)	0.75		
Parity	2.0 [1.0–2.0]	1.0 [1.0–2.0]	1.16 (0.74, 1.80)	0.52		
**Intervention**						
Received BMCFs	5 (26.3%)	70 (51.1%)	0.34 (0.12, 1.00)	**0.05**	0.24 (0.07, 0.86)	**0.03**

* Data are presented as mean ± standard deviation, number (%), or median [interquartile range]

† Statistically significant *P*-values are bolded

‡ Multivariate binary logistic regression adjusted with gestational age, birthweight, 1-min APGAR, and received BMCFs (Hosmer and Lemeshow test *P*-value: 0.56)

*BMCFs* breast milk cell fractions, *C-Section* Cesarean-section, *NVD* normal vaginal delivery, *OR* odds ratio

## Discussion

This randomized controlled trial has shown a significantly lower mortality rate in neonates with a birthweight of ≤1800 g who received BMCFs on the first day of life (OR: 0.24; 95% CI 0.07, 0.86) after adjustment with possible confounders. In subgroup analysis in neonates with a birthweight of < 1500 g, the mortality rate was more than three times lower in the BMCFs group (4 [9.5%] vs. 13 [30.2%]; OR: 0.24; 95% CI 0.07, 0.82). In addition, there was a trend for a lower incidence rate of NEC, less positive blood cultures, and shorter intubation periods in neonates in the intervention group. However, the difference was not significant in any major complications.

Despite the increased survival rate of premature neonates in recent years, some complications are the most leading causes of death [26]. As one of the most severe complications in preterm infants, NEC has a mortality rate ranging from 15 to 30%, followed by an increased risk of low long-term growth and neurodevelopmental impairment in survived infants [27]. In recent decades, many discoveries have been made about breast milk cell fractions. Breast milk contains a myriad of cell types, including leukocytes, epithelial cells, stem cells, and probiotic bacteria [4, 16]. In a study by Indumathi et al. [28], after identifying the cell surface markers in human breast milk, myoepithelial progenitors, immune cells, growth factors, and cell adhesion molecules were demonstrated the major constitutes of breast milk cell fractions. Some studies have presented mesenchymal stem cells as the most multipotent stem cells in breast milk [17, 29]. These cells can potentially differentiate into chondrogenic, osteogenic, adipogenic cells and can differentiate into astrocytes and oligodendrocytes as well as neurons [17–19].

Our study demonstrated that early use of extracted BMCFs during the first 6–12 h after birth independently reduced the risk of in-hospital mortality in neonates with a birthweight of ≤1800 g (OR: 0.03; 95% CI 0.07, 0.86), while other major complications including NEC, BPD, and positive blood culture were similar between groups. In a study by Modi et al. [10], 131 neonates with a birthweight of 750–1250 g were evaluated for early enteral feeding. All-cause mortality was lower in infants with early aggressive feeding regimes than routine regimes (33.3% vs. 43.1%; *P*-value: 0.25), but there was no significant difference in mortality or major morbidities. They have found that neonates with early aggressive feeding regimes reached full enteral feeding 3 days earlier (*P*-value < 0.01). In another study by Salas et al. [12], they investigated early feeding in 60 preterm infants (< 28 weeks). Early progressive feeding reduced the need for parenteral feeding, while in-hospital outcomes, including mortality or NEC, were similar between groups. These differences may be explained by; a) we exclusively used their own mothers’ breast milk rather than any formula. b) in this new method, precipitated BMCFs and 0.1–0.2 mm of lower semi-solid (watery) part and cellular strings were extracted and used. In comparison, other studies used the whole components of breast milk. c) different inclusion criteria regarding gestational age and birthweight can potentially affect these studies’ results.

We found a lower hospital length of stay in the control group (26.3 ± 21.9 days vs. 29.8 ± 23.8 days) which can be explained by a higher mortality rate in this group. The discharge weight was higher in the control group compared to the intervention group (1710.8 ± 229.7 g vs. 1679.1 ± 195.2 g), which can be attributed to a higher rate of formula milk consumption (14 [17.3%] vs. 6 [8.0%]; OR: 0.42; 95% CI 0.15, 1.15) and duration (18.5 ± 13.4 days vs. 14.8 ± 11.4 days) during the hospitalization in the control group while gestational age, birthweight, and sex were similar between the two groups. We observed no association between infant formula consumption and in-hospital mortality since all deceased infants were only fed with their own mother’s breast milk during their hospital course.

We would like to emphasize that our study has some limitations. First, the low sample size and lack of double-blinding may have influenced the study results. Second, the prescription of only a single dose of BMCFs in the intervention group is another limitation of this study. The repetitive use of BMCFs on the first days of life may change these research results, especially in infants who are not allowed to start enteral feeding due to an underlying disease. Third, lacking information regarding cause-specific deaths in each group is another limitation of this trial. Fourth, since it is a single-center study on the Iranian population, further multicenter studies on different ethnicities are needed. Nevertheless, according to our knowledge, this is the first randomized clinical trial that prescribes BMCFs to neonates with a birthweight of ≤1800 g. We hope this new method is followed by further researches with a larger sample size and repetitive use of BMCFs on very-low-birthweight infants.

## Conclusions

Our research demonstrated a significantly lower mortality rate in neonates with a birthweight of ≤1800 g who received breast milk cells on the first day of life. Since this is a novel method with minimal intervention, we are looking forward to developing and evaluating this method in larger studies with more frequent use of breast milk cell fractions.

## Data Availability

Data are available upon a reasonable request to the corresponding author.

## Abbreviations

BMCFs: Breast milk cell fractions
BPD: Bronchopulmonary dysplasia
C-section: Cesarean section
NEC: Necrotizing enterocolitis
ROP: Retinopathy of prematurity
NVD: Normal vaginal delivery
